# Comparison and Efficacy of Synergistic Intelligent Tutoring Systems with Human Physiological Response

**DOI:** 10.3390/s19030460

**Published:** 2019-01-23

**Authors:** Fehaid Alqahtani, Naeem Ramzan

**Affiliations:** 1 School of Computing, Engineering, and Physical Sciences, University of the West of Scotland, Paisely PA1 2BE, UK; naeem.ramzan@uws.ac.uk; 2 Computer Science Department, King Fahad Naval Academy, Jubail 35512, Kingdom of Saudi Arabia

**Keywords:** electroencephalogram, electrocardiogram, human–computer interaction, Intelligent Tutoring Systems, physiological signals

## Abstract

The analysis of physiological signals is ubiquitous in health and medical diagnosis as a primary tool for investigation and inquiry. Physiological signals are now being widely used for psychological and social fields. They have found promising application in the field of computer-based learning and tutoring. Intelligent Tutoring Systems (ITS) is a fast-paced growing field which deals with the design and implementation of customized computer-based instruction and feedback methods without human intervention. This paper introduces the key concepts and motivations behind the use of physiological signals. It presents a detailed discussion and experimental comparison of ITS. The synergism of ITS and physiological signals in automated tutoring systems adapted to the learner’s emotions and mental states are presented and compared. The insights are developed, and details are presented. The accuracy and classification methods of existing systems are highlighted as key areas of improvement. High-precision measurement systems and neural networks for machine-learning classification are deemed prospective directions for future improvements to existing systems.

## 1. Introduction

Modern technological progress has paved way for human–computer interfacing research. The human brain is a very complex system. There have been diverse attempts to observe, understand, and model behavior based on human response to stimuli, based on either empirical understanding developed by psychology [1,2] or by use of biotechnological equipment such as Magnetic Resonance Imaging (MRI) and Electrocardiogram (ECG), by measuring blood and oxygen flow patterns to the cerebral region, also known as the hemodynamic response [3]. Both the empirical and machine-based methods are used together in modern systems for a thorough understanding of human patterns and brain activities, to determine how a human will respond to a stimulus, which can be quantitatively observed and processed with appropriate equipment. The physiological response of human beings falls under the umbrella of behavioral neuroscience and is largely dependent on their emotions. It is interesting to note that the body’s response to stimuli can be quantified since the response patterns occur either in the Central Nervous System (CNS) or the Autonomic Nervous System (ANS) [4]. These responses patterns cannot be spoofed or controlled by the person consciously, while subjective visual assessment or tone of voice also known as audiovisual assessment, can vary between individuals of different genders, cultures, and ages. It is a major reason for the lower accuracy of subjective and non-physiological-based methods [5,6] compared to the physiological assessment-based techniques. In contrast, the ANS responses have been found to be similar in people across continents in a study conducted by Levenson [7]. It was found in [7] that the human ANS reacts similarly for negative emotions such as sadness, anger, and disgust, despite having cultural and regional differences. Emotions are reported to be distinctively classified into various forms, such as Ekman et al. [8] proposed six basic emotions, while Parrot [9] presented tree structure of emotions. On the other hand, valence-arousal scale by Russell [10] and Plutchik’s emotion wheel [11] identifies the dimensional scale of emotions. Russell’s valence-arousal scale is extensively used in applications to quantitatively relate emotions. The arousal and valence are liable for the variation in emotional states and can be placed on horizontal and vertical axis on a 2D plane, respectively. The emotions can be categorized effectively by using the dimensions of dominance in the model, proposed in [10].

In published research, ITS have been reviewed based on their performance within both theoretical and practical scenarios. However, ITS being a broad descriptor, means that there is a large variety of techniques towards the same goal. For instance, Kulik and Fletcher (2016) [12] studied the effectiveness of 50 different ITS in the areas of reading comprehension, algebra, arithmetic and more, and statistically analyzed the mean results of improvements, noting up an improvement in students from 75th percentile to 50th percentile. The review concluded that in the scenario of teaching by ITS followed by an evaluation, the improvements in evaluated score depend upon the nature of the ITS as well as the quality of ITS in question. A review by Ma et al. [13] suggests that several studies between 1997 and 2010, place the learning outcome of ITS help more with course-specific evaluation than generalized testing. They also suggest that quality of reporting ITS results is lacking in modern literature, and a meta-analysis approach shows that while ITS has shown to be effective in key areas compared to conventional tutoring, it is not yet ready to totally replace standard practices. A 2015 review by Nye [14] highlights some barriers in ITS implementations globally, citing the problems that while ITS is suited to developed countries, it is a challenge to initiate in the developing world. Nye also states that a problem in published ITS research shows a selection bias, as the systems are mostly implemented and tested in environments best suited for them, with large resources and stable electricity provision, etc.

Ajuha and Sille [15] show the gradual improvements in an ITS architecture and conclude that the ITS methods have been gradually transitioning into individual-level personalized teaching tools. Malekzadeh et al. in [16] review the importance of adding emotion recognition into an ITS’ architecture, since it takes into account the personalized needs of the student. Furthermore, their work recognizes the emotion recognition aspect as a core component of an effective ITS. Despite the shortcomings and challenges, ITS is a promising area of technology. There are confirmatory results that ITS does not have a negative impact on K-12 level [17] or college level learning [18], making it an effective tool for use in the classroom.

With continuous progress being made in modern tutoring methods and a focus on effective pedagogy using technology, it is important to keep updated with the evolution of ITS. For this purpose this paper includes the overview of currently used models in research as well as the results achieved by recent papers. The main contribution of this paper is that it presents an amalgamated structure of information of ITS, as well as the popular measurement techniques used in modern methods. We present the advantages and disadvantages, as well as the requirements of a modern system based on principles of human–computer interaction (HCI). Furthermore, pros and cons of various algorithms are discussed in detail. This paper aims to cover a broad range of topics which includes results of experimental testing and feasibility of various approaches taken by recent research contributions.

The layout of the paper is as follows. First, an overview of ITS why it is necessary, and its characteristics are presented. The currently available and viable methods of measuring physiological signals are discussed, followed by a comparison of the various implementations available in literature. This is followed by a section which discusses at length the techniques of emotional classification and machine implementations of these classifiers. The consequent section details the significance and uses of a modern ITS and presents a comparison of ITS tools available and their purpose. Finally, conclusion is presented with directions on future work.

## 2. Intelligent Tutoring Systems (ITS)

In terms of learning, technology has made vast progress in aiding humans to tutor and acquire new skills. The main forms of tech-based information conveyance are; (1) audio-based information, such as radios, audio tapes and phonographs, (2) visual/video which includes presentations, television, documentaries, and films, and (3) combination of audiovisual methods such as PC and mobile phone applications [19] or software. There is a rapidly growing number of universities that offer what is known as technology-mediated tutoring whereby courses and learning tools are offered through the Internet or dedicated applications. Past research [20] has investigated the role of interactive electronic tutoring in the classroom. Instead of simply offering data and resources to learners, the idea was to present tutoring methods that have real-time participation by the students. It was previously established that interaction plays a vital role in learning, and can drastically increase the quality of learning [21]. Further research has built itself on the idea of interaction, since an active participation allows the tutor to obtain important feedback and adapt changes in the tutoring method [22].

In Computer-Based Training (CBT) systems, computers deliver the instruction via some computer program(s) known as software. In literature, CBT is also recognized as Computer-Based Learning (CBL) or Computer-Based Instruction (CBI). CBT is a large set and ITS are a subgroup of CBT. The major difference between CBTs and ITS is that CBTs deliver tutoring continuously to all students/learners, while ITSs are “intelligent”. Based on triggers, ITS systems are highly adaptable in real-time scenarios. In ITS, triggers are learners’s state(s) or the training settings. The “production rules”also knows as policies, are usually used by an instructor to identify the changes and learning outcomes.

There have been many applications of these technologies historically, in categories of language learning [23], and it has been shown from studies that the use of mobile phones to provide quizzes, translations, and practice words from applications were not effective in learning new content due to the small screen size and often bad ergonomics; however, they were effective for reviewing and taking short practice quizzes [24]. It has also been stated from reviewing experimental studies with novel tutoring technologies, that the use of mobile phones and computers in pedagogy in an inevitable future, whereby techniques such as Mobile Assisted Language Learning (MALL) will become the de-facto methods of language tutoring and practice [25]. Besides language learning, technology has also played a role in learning new motor skills and enhancing the learning prospects of people with disabilities [26].

Intelligent tutoring—by definition—involves the use of computers. However, to establish the benefits of intelligent tutoring one must compare it to other types of tutoring methodologies to determine any advantages. Human tutoring, as discussed, has been the forefront of learning since the dawn of formal education, and even before. We can define a human tutor as an adult, who is reasonably experienced in the study of the subject, and works with one or multiple students to impart some knowledge or skill, in person.

**Figure 1:**
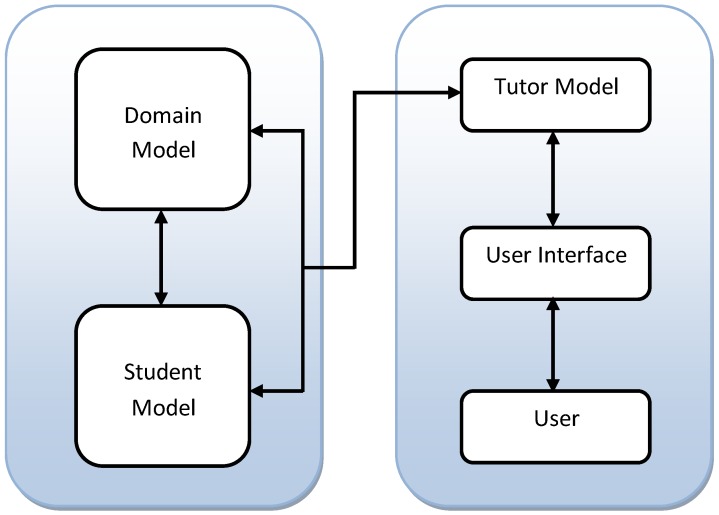
Architecture of an ITS.

In contrast to this, we can define computerized tutoring in one of two ways [27]: (a) The use of Computer Aided Instruction (CAI) which involves giving a problem to a student and asking for an answer; and depending on the answer’s correctness the computer asks the student to try again or gives marks accordingly; or (b) providing the student with a user interface with multiple functions, such as a form, or an audio dialog, with worded problems and the student is asked to solve the problem step-by-step on the digital medium—referred to as an ITS. An architecture of a generic ITS is shown in Figure 1.

**Figure 2:**
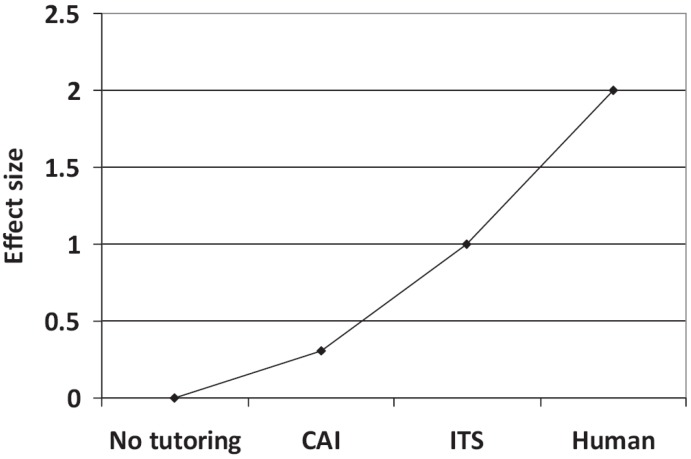
Graphical representation of commonly believed effect/impact vs tutoring methods.

It is stated in past research that human tutoring is more effective than computerized methods, and is believed to have the highest impact on learning [28,29]. A graphical representation of this can be seen in Figure 2. This conception is attributed to a series of hypothesis which include fast adaptation to student behavior, better control over dialog/situation [30], selecting tasks more suited to the individual student’s needs, motivation [31] and application of tutoring strategies which may not be simple to implement in a computer.

**Figure 3:**
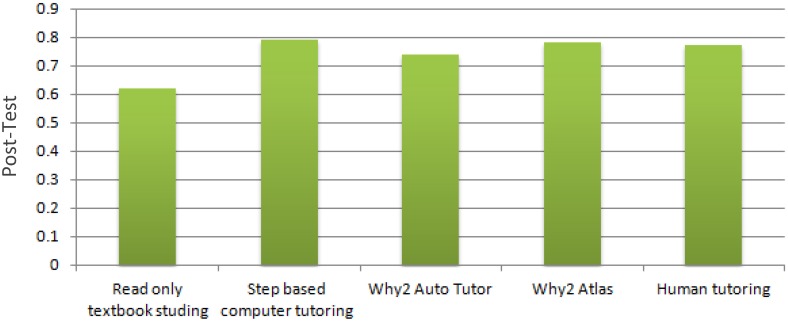
Different type of ITS [32].

However, a study by the Van Lehn [32] disproves the notion of objective superiority of human tutoring, by comparing student learning in the subject of physics. The study was conducted such that the questions posed to the students were step-based, i.e., the conclusion was arrived at by answering a series of questions. The scenarios compared were textbook reading (i.e., no tutoring), a step-based computer tutoring system (CAI-based), Why2-Atlas (which is an automated physics tutoring software with granularity [33]), Why2-AutoTutor (which is another physics dialog-based automated ITS [34]), as well as human tutoring. The results, shown in Figure 3 show that the systems are on par with human tutoring, as shown by their impact/effect. This leads to the feasibility and importance of ITS, and why through properly refined software algorithms it can lead to an effective learning solution.

There are various methods which can be used to determine human responses and emotions. These human emotions can be further used in ITS. The next section discusses well-known Electroencephalography (EEG) and Electrocardiography (ECG) and contactless measurement methods for ITS.

## 3. Measurement Techniques

This section discusses some measurement techniques such as Electroencephalography (EEG) and Electrocardiography (ECG) and its uses in human emotions responses. The contactless measurement methods such as facial analysis as a human response are also explained. The use of the aforementioned human physiological emotions and modeling techniques used in ITS is explained in this section.

### 3.1. Electroencephalography and Electrocardiography

There also exists literature for the use of Electroencephalography (EEG) in measuring human emotional response [35,36,37], whereby the brain signals of the participants were used to classify emotions and emotional responses. One of the studies [35] states that the overall response is due to two sources; the peripheral signals, and the ones obtained from EEG. EEG is used to determine solution(s) which is closely associated with the electrical activities of brain [5]. An EEG tracks real-time brain signals in the form of voltage changes which is then send to computer software for further processing. A normal activity of brain is a recognizable pattern and abnormal/different patterns can be used in ITS applications. In 1924, Hans Berger was the first researcher who reported EEG in human [5]. There are numerous relations between EEG and psychological phenomena. However, a precise and exact link between both are not completely understood.

Another popular method to determine the effects of stimuli or learning on the body is Electrocardiography (ECG). As a non-invasive and easy to use method, it has gained quick popularity in non-medical uses. The technology is now at a mature stage, due to over a century of research in the area [38]. Other advantages of ECG include the lower cost of equipment, standardization, ease of use and widespread availability. A commonly used metric obtained from ECG is the heart rhythm variability, commonly known as HRV, which has found uses in the field of psychology and the related aspects of computational intelligence. An HRV signal enables us to see the physiological response of a psychological stimuli [39], although the relationship is complex and does not always guarantee results. The HRV response has been used to understand the relationship between the sympathetic and parasympathetic reflexes and responses, which are the most basic human responses to any situation, with the former responsible for the fight-or-flight reflex, and the latter dictating the state of relaxation and receptiveness of the body [40,41]. Past studies performed on heart rate and HRV show that the heart is not consistently periodic under normal conditions, even in the absence of stimuli [42] and rather shows a non-linear response in resting condition. This has led researchers to investigate new methods such as non-linear dynamic theory towards modeling the subtle changes in heart rate time sequences, such as approximate entropy (ApEn [43]—a model independent technique which detects patterns and determines the difficulty of future prediction), correlation dimension (in terms of application of Chaos theory as a correlation/prediction model on pathological signals) and Hurst exponent (long-term dependence and correlation between two time series) [44].

**Figure 4:**
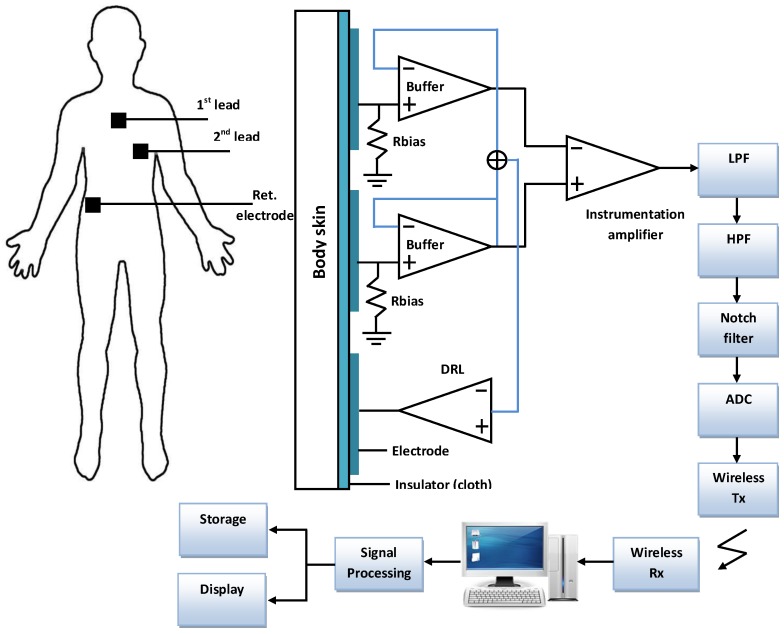
ECG probe placement and measurement devices [45].

**Figure 5:**
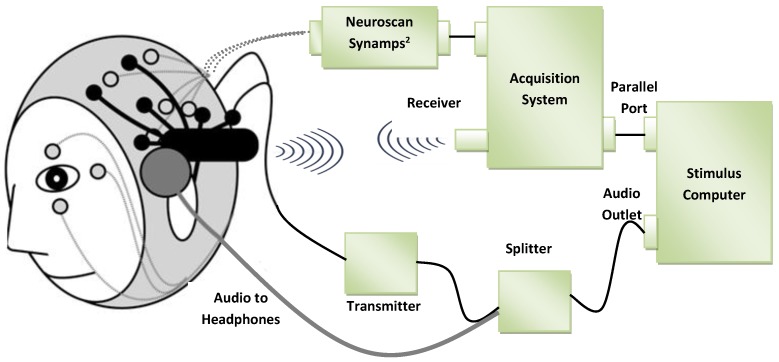
Connections and measurement via an EEG device [46].

Measurement methods of both ECG and EEG are shown in Figure 4 and Figure 5, respectively.

### 3.2. Contactless Measurements

In recent literature there is a growing focus on facial analysis as a form of non-invasive means to measure physiological signals. This method also began with medical applications, and focuses on computer-vision based tools to analyze the expressions of a patient during check-ups, treatment, and rehabilitation or therapy. For instance, if a practitioner notices pain or difficulty in the patient’s expression, they may stop or slow down the exercise. Therefore, facial recognition has been cited as one of the more important aspects of estimating psychological, physiological and physiological indicators in a subject [47]. This method provides a contactless method to obtain signal data, which allows for a more comfortable experience for the subject. An extension of this method, built upon the same contactless measurement philosophy, is termed photoplethysmography (PPG), which uses an infrared light on the part which is to be evaluated, and the reflected beam is assessed for indicators. Variations in the reflected beam are used to check for changes in heartbeat; however, this method is susceptible to motion sensitivity [48]. There is also literature on implementation of non-invasive sensors in the seat occupied by the subject to measure heart and respiration rate, and the resulting data was found to be within 5 bmp for resting respiration rate and in the range of 15 bpm resting heart rate [49].

**Figure 6:**
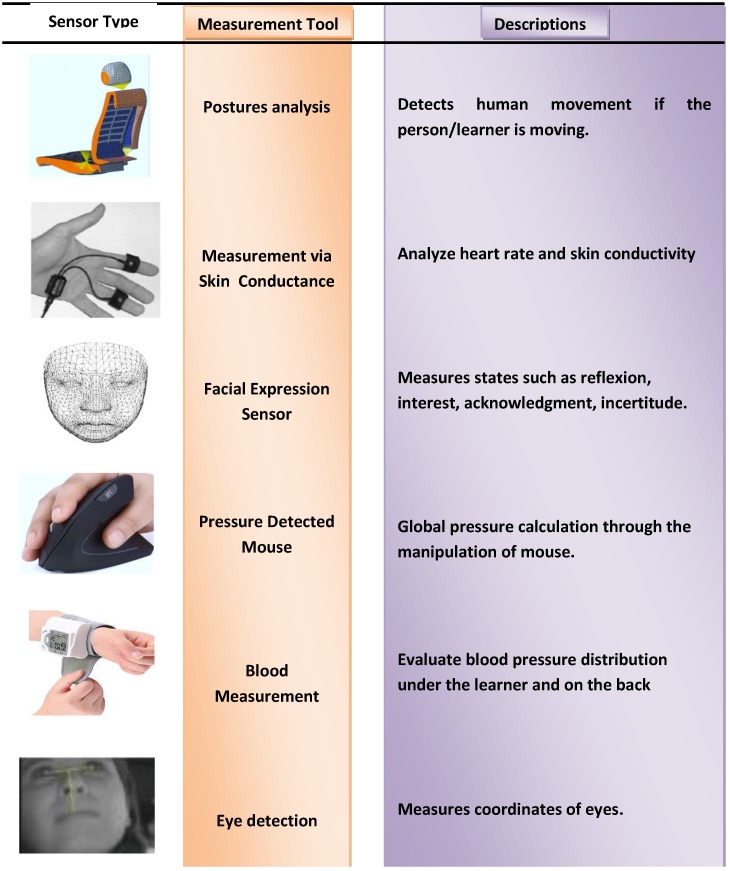
Various other measurement and contact-less devices [51].

The comfortable nature of contactless and non-invasive methods, coupled with the improvements in the accuracy of modern sensors, is showing continuous improvements and innovative means of obtaining and processing psychophysiology signals. In fact, with the use of infrared imaging alone it is possible to read a subject’s blood perfusion rate, breath rate, stress response, etc. [50]. Various types of sensing methods including contactless methods such as eye detection are shown in Figure 6.

### 3.3. Performance Analysis

A detailed analysis obtained by various researchers using the aforementioned measurement techniques, (ECG and EEG, etc.) are given in Table 1.

### 3.4. Physiological Modeling and Classification

#### 3.4.1. Valence-Arousal Model

A well-regarded ‘valence-arousal’ model exists in which emotions are classified according to their valence and arousal levels [62] with the classifications being negative/high arousal (anger, disgust), positive/high arousal (happiness, excitement), negative/low arousal (fear, mournful) and positive/low arousal (relaxation, surprise). In a study based on the valence-arousal model [53], the author designed and used an automated statistical search-based classification system for the physiological signals obtained from ECG, skin conductivity changes, and EEG. A classification accuracy of up to 95% was achieved with this method. Another study [63] shows the applicability of the ECG in classification of emotions in terms of sadness, anger, stress, or surprise, depending on the kind of the kind of stimuli provided, such as the subject being told a sad story, or the subject pressured into completing a very difficult task in a short time span, or by surprising the subject with a loud noise. For the sample of 50 subjects, the 78.4% for three emotion classification and 61.7% for four emotion classification, which is much higher than chance probabilities.

Experimental studies have shown the effects of drugs and stimuli responses in suppressing and enhancing the effects of these two aspects of the ANS due to variations in the arterial pressure. It was also observed that the cardio response to these effects is not only causal, but also causes a certain feedback, e.g., the lowering of heart rate triggers the sympathetic system to stabilize and increase heart rate [64], thereby establishing that the two are not just reciprocal, but also interconnected and the overall body response is a combination of factors through the ANS. It has been concluded from cardiovascular responses that certain high-stress activities such as public speaking or giving an exam can cause high-stress-related hemodynamic responses in the body, with the heart function adjusting accordingly. Two types of cardiovascular responses have been noted, namely, vascular resistance (the ease or difficulty of blood flow through the system) and heart output in terms of beats, also known as myocardial response [65]. This is based on previous research by Obrist [66] which investigated how active tasks trigger a vascular response and passive tasks trigger a myocardial response. However, this response is not just ‘either-or’, and a combination of vascular and myocardiac responses can be observed depending on the individual’s coping style to stress and the stressor activity at hand [67]. For instance, in a study where participants under observation was subjected to the stressor of public speaking, it was noted that before and during the presentation, the response was more myocardiac instead of vascular, despite it being an active task [68].

A study focused on stress effects on HRV, and the effects of the work and rewards was conducted [69] where a group of participants were monitored periodically over a span of three days. The observations were performed on the vagal tone (via ECG). It was noted that in the participants who did not achieve rewards consistent with their effort had a perceptively bad mood, which was reflected in the obtained vagal tone readings. It has been proven that a negative attitude or bad mood is linked with reduced learning performance and lower cognitive process [70] while rewards and achievement systems aid in cognitive improvements [71]. It has also been shown that human emotions play a significant role in learning and rational decision-making and learning [72]. It makes sense, therefore, that the ECG has been investigated in research focused on learning improvement.

#### 3.4.2. The Stein and Levine Model

The relationship between individual emotions and goals was identified by Stein and Levine [73] through goal-directed and problem-solving approach. In contrast to other models (happiness/sadness), their model expects that all individuals attempt to integrate new and unfamiliar information into current schemas, which are packages of world knowledge, such as stereotypes, scripts, frames etc. Stein and Levine [73] also assumes that emotional involvement is closely related with incoming new information. New information causes arousal of the autonomic nervous. Arousal of the ANS occurs due to the mismatch between available incoming novel information and existing schemas. The conjunctional occurrence of ANS arousal and cognitive estimation of the situation always causes emotional reaction. This model predicts that learning almost always takes place throughout an emotional state.

#### 3.4.3. The Kort, Reily, and Picard Model

A four-quadrant model known as Kort, Reily and Picard model, clearly relates learning and current states. In the aforementioned model, the learning process was broken into horizontal and vertical axes which was tagged as affect and learning, respectively. The horizontal axis i.e., learning axis ranges from “constructive learning” at the topmost and “un-learning” at the bottommost. The new data or information is being combined into the schemes and fallacies are mostly identified and removed from the schemes. The vertical axis (affect axis) ranges from positive effect on right to the negative effect on left. Depending on learners starting points, learners can move around the circle in different patterns. According to this model, learner can move from an easy state, to encountering fallacies, to discarding fallacies, to new knowledge, and then back into easy state. In this four-quadrant model, the time spent in each quadrant is dependent on numerous factors. Further studies on cycling procedure via these emotions during learning is now being explored in a paper [74].

#### 3.4.4. The Cognitive Disequilibrium Model

There exists a class of cognitive models that hypotheses a significant role for cognitive disequilibrium in learning procedures and comprehensions. Deep comprehension results in, when learners encounters peculiar events, disagreements, hindrance in goals, disruptions, revelations, identical alternatives or experiences which do not meet the desired expectations. Cognitive disequilibrium has a high probability of activating conscious, intellectual considerations, questions and queries aiming to reconstruct cognitive equilibrium. The emotional states of frustration and confusion are most likely experienced during cognitive disequilibrium. Literature review indicate that confusion is one of the most important emotional states as far as scientific studies are concerned. The confusion is a state where a person cannot thing clearly, having hard to make decisions or is completely disoriented. Moreover, confusion is usually followed by cognitive disequilibrium. On the other hand, the state of hesitation and disturbance show that more information and interpretation is required.

### 3.5. Classifications of Emotions

Classifying and evaluating affective emotions states requires some computational efforts. During a tutorial dialog, there are a set of input features $I_{F}$ and a set of classifiers {*C*_1_, *C*_2_, …, *C_n_*}. A specific classifier $C_{i}$ is generally dependent on some input features $G(I_{F})$. The classifier $C_{i}$ is then predicted through $G(I_{F})$. In many case studies, classifier is the combination of standard and biologically inspired classifiers. Emotion classifier is driven through the aforesaid two combinations of classifiers. The standard classifier can integrate the obtained data to a high-dimensional vector and the biologically motivated/inspired classifiers individually classify each channel. Lastly, the classification of every channel is combined and the output as a single emotion is determined. Each method has their own advantages and disadvantages and reliably finding which approach is highly accurate is an empirical thing. The output of each channel is generally independent and hence it is difficult to conclude that which classifier is optimal. Therefore, a serious of different or combination of various classifiers are used in emotions states classification for getting the best optimal classification.

#### 3.5.1. Standard Classifiers

Standard classifiers are widely used due to their reliability and because they have been repeatedly implemented with acceptable results. They can be divided into broad categories such as Bayesian Classification, Decision Tree method, Fuzzy Classifiers, Neural Networks, and Genetic Algorithm Classification, etc. [75]. Each of these categories in turn consists of multiple specific types of algorithms. These classifiers hold the advantage of being implementable and computationally feasible and can therefore be considered in future work of emotional classification.

#### 3.5.2. Biologically Motivated Classifiers

In last decade, some researchers have highlighted the dynamic behaviors of neural populations. In their studies, higher level brain processes were closely examined through fine grained analysis of neural populations. Freeman and his associates developed a biologically inspired model known as K-set. This model repeats experimentally recorded data of the dynamic behavior in the olfactory system [76]. Experiments for this model can be performed which can be further tested through simulating spatial and temporal properties of neural populations. The KIII network basically work as a classifier which produces low-dimensional local basins of attraction. Difficult classification problems are solved via such low-dimensional local basins of attraction when data cannot be linearly separated. This network basically serves as an interface among standard classifiers and noisy data. Moreover, the KIII network learns through three methods, Hebbian reinforcement, habituation, and normalization. The KIII network has been thoroughly validated via detailed experiments and then reported as a most accurate classifier in [77]. Synthetic and real-time experiments proved the optimum behavior of KIII. Detection of small machine parts, and classifying EEG patterns, the aforementioned model was highly accurate.

## 4. Use of Human–Computer Interaction

A computer system which understands the emotions of human and facilitates the people in achieving their goals efficiently and safely is called HCI system. In learning and decision making, human emotions play a vital role. HCI make a life easier for humans in performing different tasks. For example, knowledge of the emotional state of a patient would be helpful to a psychiatrist in the diagnosis of a mental disease. Similarly, a new born baby who cannot express his emotions may be difficult to diagnose due to lack of verbal communication. However, with the help of computers in understanding emotions and psychological signals will make it easier to diagnose the disease [72,78,79]. Over the past decades, there has been heightened focus on research involving emotions recognition. As stated previously, are many different modalities to recognize human emotions, such as audio, body language, facial images and psychological signals [80,81]. In recent times, considerable works have been done in emotions recognition using psychological signals. Besides EEG and ECG/HRV already discussed, other physiological signals used in Human Computer Interaction (HCI) studies are: Respiratory Sinus Arrhythmia (RSA), Electromyography (EMG), Galvanic Skin Response (GSR) and Electro Dermal Activity (EDA) [82,83].

To gather and understand the psychological signals, an interface is to be needed between humans and computers. Interfacing techniques have been improving day by day and research work in this area has been growing over the past few years. The already existing physical technological devices are designed for HCI can be classified on basis of three human senses: vision, audition and touch [84]. The devices based on vision are most common in practice; they are either switch-based or pointing devices. Switch-based interface devices have buttons and switches like keyboards. Examples of pointing devices are joysticks, graphic tablets, touch-screen panel, and pen-based input. The devices based on audition are more advance and complex in nature. These audition-based devices are very useful in interaction but on the other hand very difficult to build [85]. Haptic interfacing devices are based on human sense, i.e., touch. After touching with skin and relative rigidity with muscles, these devices generate different sensational signals. Haptic devices, however, are very costly and complex to build; these are manufactured and used for virtual reality and disability assistive applications [86,87,88].

The modern interactive technologies in HCI are combining existing interactive methods with other advanced technologies such as networking and animation. These new modern devices can be classified in three sections: wireless devices, virtual devices, and wearable devices. These devices are being used in multiple fields including smart healthcare [89,90,91,92]. Some of the examples of these devices are: RFID tags, GPS navigation system and some other GPS-based tracking system [85]. For better understanding of data, a robust and real-time interactive system that can give noise- free psychological signals is vital. In designing of emotions recognition system, all these modern devices are being used. In [93], quality of video was evaluated with the help of physiological signals. Wireless EEG headset, Shimmer wireless ECG, and Shimmer wireless EMG sensor were attached to human subject. ECG, EEG and EMG signals were recorded for further processing.

Once data is collected through interfacing devices or sensors, data is pre-processed. Pre-processing is very essential step in emotions recognition systems. The physiological signals are always adulterated with extreme noise [62]. In addition to noise, other external and internal interferences caused by electrostatic devices effect the raw signals [93]. Different tools, methods, and software are used for elimination of noise and interferences from physiological signals. Augsburg Biosignal Toolbox was used for pre-processing of EMG signals in [94]. EEGLab specialized toolbox was used to eliminate noise from EEG signals [95]. The noise from Facial EMG signals was removed with the help of Low-pass filter. General types of Low-pass filters are: Elliptic filters, Adaptive filters and Butterworth filters [62]. Smoothing Filters were used to eliminate the noise from raw Galvanic Skin Response (GSR) signals [96,97]. ECG signals are less liable to be influenced by noise and interferences unlike EEG and EMG because of higher voltage amplitudes. Therefore, no pre-processing is needed for ECG signals [93]. Once pre-processing is done, for classification of signals different features are extracted from each processed physiological signal. Extracted data is used in detection of emotional contents in respective signals.

Different features which are extracted from various processed psychological signals are: statistical data, time domain, frequency domain and time-frequency domain. In [53], one hundred and ten features were extracted using ECG, EMG, Respiratory and Skin Conductance signals. The main features that were extracted in this study are geometric analysis, statistics in time series, power, multi-scale sample entropy, frequency domain, sub-band spectra etc. Many different methods have been used so far in extraction of features, such as Fourier Transform, Hilbert Huang Transform (HHT), Wavelet transform, Robust Singular Spectrum Transform (RSST), Empirical Mode Decomposition (EMD), etc. [62]. A Fourier Transform is used to decompose the time-domain psychological signal in frequency domain [98]. To get the instantaneous frequency features of time-based physiological signals along with intrinsic mode functions HHT is used. Contrary to the Fourier transform, Wavelet Transform has ability to transform a signal into time-frequency representation by dividing continuous time-based signal into wavelet [99]. Mel-Frequency Cepstral Coefficients (MFCC) and Kernal Density Estimation (KDE) were used in extraction of statistical features for EEG and ECG.

In [93], EEG-based features were extracted using power densities (PSD) of different bands of frequencies. PSD of each band of frequency is used to identify the patterns in EEG signals. Welch power estimator was used to compute the power spectral densities [100,101]. In ECG signal, various kinds of features were extracted. Firstly, Augsburg Biosignal Toolbox (AuBT) was used to identify the PQRST complex of ECG signals [94]. By deducting time elapsed for the correlating pairs of peaks of each ECG signal, PQ, QS and ST complex were extracted. For EMG signal, statistical features were taken out from respective signal and as well as from its first and second derivative using AuBT in the same study. These statistical features included minima, maxima, median, mean, standard deviation and number of times per unit time EMG signal was reached [93].

Some features from various psychological signals may or may not be correlated with human emotions and can be removed [62]. Researchers have used Various Algorithms for features reduction. Some of these algorithms are: Fisher Projection, Sequential Forward Selection (SFS), and Sequential Backward Selection (SBF) etc. [72,102]. Based on these finalized features, emotional states are classified [103].

## 5. Modern Intelligent Tutoring Systems

Modern ITS methods implement the psychological learning behaviors discussed above, as well the technological aspects of learning to create effective tools which have shown to be a drastic improvement over old methods. Used properly, ITS has proven to be a very effective form of learning.

There are a variety of tasks expected from a modern ITS that define its effectiveness. One popular qualifier for effectiveness of any chat-based computer software is the Turing test, developed in 1950 by Alan Turing [104], which is to determine whether a machine could ‘think’, i.e., whether it could converse with a human in a human-like manner (termed the imitation game), such that the human would not be able to differentiate whether the conversation was being carried out by human or software. The earliest techniques for imitating this behavior were developed with the system ELIZA [104] which used a system of pattern matching, such that any sentence provided to the bot would be matched against a set of strings with keywords and the answer would be generated [105]. Other software that use pattern matching include Jabberwock by Pirner, 2005 [106] and HEX by Hutchens, 1997 [107]. In addition, authors such as Abdul-Kader [108] and Bardesko [109] have pointed out that chat programs are often equipped with tricks such as deliberate typing mistakes and short responses to make the user believe they are conversing with another human. Masche and Le discuss in [105] the common components in a modern ITS are a pre-processing code, which delegates the context awareness duty to the Natural Language Understanding section, while a separate Dialog Manager is responsible for keeping the conversation flowing. The dialog manager also checks which keywords in the phrase to base the response on, from a pool of phrases and words that are pre-programmed conversational sentence structures. The response which is sent back can be generated from a variety of ways, as highlighted by Masche and Le in [105] such as a separate generation module, a natural language generation module, or a combination of multiple components.

ITS systems are based on these chat-based systems, often using a direct conversation approach with the student. A good ITS requires adaptability and as close to a human response as possible such that the student’s emotions and behavior are quantified and the learning adjusted accordingly. The feedback systems such as AutoTutor demonstrated by Graesser et al. [110], and the GnuTutor-based feedback system described by Ivanova [111] are built upon the ideas of these adaptable chat systems and are capable of assessing student mood to adjust the output and teaching style accordingly.

One method of understanding student behavior is known as the ICAP (Interactive Constructive Active Passive) method proposed in [112], which classifies behavior in order of highest effectiveness to lowest:

interactive≥constructive>active>passive.

The idea is to assess the student during the learning to see where, on the spectrum of ICAP scale, the student engagement level is. The system is defined as follows:

- Passive learning is where the student is listening to or receiving the teacher’s instructions, without displaying any overt behavior that signifies high intellectual engagement. It is the classified as having the learn receptiveness to learning.
- Active mode is defined as the student actively taking some action in the learning, such as choosing one out of the presented options or pointing/gesturing to their answers. Active learning is classified as a higher learning mode compared to passive learning.
- Constructive learning is where a learner contributes to what is being through by overt display of attentiveness and active participation in discussion. This can also include a student, for instance, creating a diagram of a text-based solution or giving a presentation on some idea.
- Interaction refers to a student’s ability to incorporate constructive learning with some participation or interaction with the environment, be it a group discussion or generate some knowledge that is beyond the idea taught up to that point.

**Figure 7:**
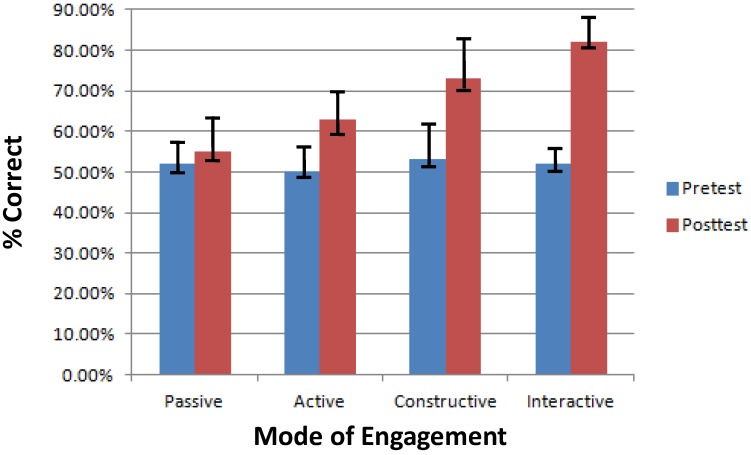
Results from [113] shows the effectiveness of interactive learning compared to other modes of engagement.

These classifications are admittedly very broad, but produce some delineation in student behavior, which can be better translated into a discrete-set of data for computer-based implementation or an ITS solution. The ICAP taxonomy helps with better understanding broader aspects of learning behavior which can be useful when creating, say, a step-based computer tutoring system that adapts to the student’s behavior and responsiveness to enhance engagement and maximize learning. A study to evaluate the effectiveness of the ICAP system [113] shows that students, when tested with a quiz before and after a test (pretest and post-test, respectively), show the highest improvement in score when taught by interactive learning. The results are shown in Figure 7.

Exposing a subject to certain learning stimuli and measuring their response through one of the afore-stated methods—while mostly involving computers or technology—is not a fully automated process as it requires human (tutor) input and supervision. Automated techniques have gained traction to circumvent this idea and remove the problem of human error in the process. In a modern ITS, the main goals are to; (a) create a user interface which enhances visibility of useful data, (b) the individual steps the student takes in the learning process or quiz are each assessed individually, (c) hints and explanations which are context-specific, and (d) presenting a problem that is tailored to the specific student. Essentially, the software or tutoring system should ‘stay out of the way’ such that the focus is on the content instead of on the system itself [114]. Out of these steps, the feedback and measurement systems are perhaps the most important to implement, as they need to work in real-time and require complex algorithms to make sense of the data being fed back.

Several example ITS tools [115] available are shown here, in Table 2.

## 6. Conclusions

This paper deals with concepts of physiological signals and how they can help determine the mental state and attentiveness of human subjects in modern ITS. Modern physiological signals, such as EEG and ECG, have expanded to uses beyond the health and medical fields, and are being used to measure human emotional responses to surrounding environments and have become a source of feedback to dedicated stimuli in environments such as ITS. With the help of computers and advances in HCI, the use of electronic signals obtained from the measurement systems has become a viable alternative for creating automated systems with suitable algorithms. These systems are capable of being trained and can be used to create non-invasive tutoring systems. In this paper, it is outlined that the current data/feedback acquisition systems and state of existing technologies provide an exciting prospect to incorporate physiological signals with ITS. It facilitates the process of feedback and data acquisition from the learner and training of systems which can automate the learning process. The intelligent tutoring system based on human emotions can adapt depending upon student mood, attentiveness, and emotional responses. As a conclusion, the synergism of physiological signals and intelligent tutoring systems can, therefore, be considered an interesting and promising venture.

To this end, the goal of further research in this area is investigating new uses for physiological signals to measure student response to teaching. For instance, experimental testing can be carried out on a group who are asked to attend a lecture and solve a test at the end to check for responsiveness, boredom, and frustration, with the use of contact-less methods such as face detection and IR-measured eye response. Contact systems such as ECG and EEG are accurate and lead to high classification accuracy. However, many subjects find them uncomfortable and they are cumbersome to place on each person individually. Some work has already been done on sensor-free detection with the proper software [117]; however, without the use of proper measurements, the accuracy suffers. With the use of modern, accurate sensors and cameras, the author believes that the accuracy of these tests can be improved with proper algorithms such as a neural network and deep learning classification approach. Standard- and biological-inspired classifiers can be used for classification and evaluation of effective emotions states in ITS.

## Figures and Tables

**Table 1:** Comparison and results of pre-existing physiological-based learning research.

Source	Measurement Techniques	Objective	Methodology	Results
Brawner and Goldberg [52]	ECG and GSR, Measured: heart rate, mean, SD and energy of GSR signal	To train bilateral negotiations with ITS, and check stress and anxiety levels via measurement during the learning process	CMT, a web-based prototype Participants interacted via static dialog for a sample size of 35	Anxiety, boredom, stress, and frustration were checked using GSR. For well-defined, no interruption learning, the average signal densities were lower by 67% compared to interrupted learning, meaning much lower anxiety and stress.
Chanel, et al. [37]	EEG and plethysmograph, Measured: arousal and blood pressure	To assess the impact of stimuli on test subject using the valence-arousal model, using mathematical classifiers	Exposure of 4 test subjects to various videos Comparison of measured values of arousal and valence to a Self-Assessment Manikin (SAM) quiz	The Bayes classifier resulted in an average accuracy of 54% between the test subject’s SAM score and the measured EEG readings. EEG signals were found to be reliable indicators with up to 72% accuracy in a test subject.
Kim and Andre [53]	EMG, ECG, GSR, and Respiration Measured: Skin conductivity, heart and breathing rate	Emotion recognition of subject based on physiological impact of music listening	Subjects listened to music and classification performed between responses on the valence-arousal model. The classification was performed using a novel emotion-specific multilevel dichotomous classification (EMDC) technique	Emotion recognition on arousal-valence was found to be up to 95% for subject-dependent classification and 70% for subject-dependent classification using the EMDC model which is higher compared to previously established PLDA classifier.
Pour, et al. [54]	ECG, EMG, GSR Measured: skin conductivity, heart rate, and facial muscle signal	Assess the correlation between physiological responses and self-reported learning by subjects when taught using a feedback-based learning software in the fields of hardware, internet and operating systems	Autotutor, a dialog and feedback-based learning software. Using Autotutor, 16 learners were asked to self-report their learning progress while being monitored for physiological signals	Using support vector machine (SVM) classifier, the classification accuracy for emotions vs. self-reported learning was up to 84% which shows the feasibility of the approach
Hussain, et al. [55]	ECG, EMG, respiration, and GSR Measured: skin conductivity, heart rate, facial muscle signals, breathing rate	Establish emotional classification for subjects in terms of categorical (frustration, confusion, etc.) and dimensional (valence-arousal) by interaction with ITS	To achieve the objective, feature extraction from data was implemented in MATLAB [56]	Baseline accuracy was established at 33%. For video measurement accuracy was up to 62% for dimensional classification and 45% for categorical classification. For a combination of video and physiological signals, accuracy was up to 64.63%
Barron-Estrada, et al. [57]	N/A	The paper focuses on analyzing the sentiments of the learner and implement the results in an ITS.	Sentiment Analysis Module, a computer-based system, used to take subject opinions in terms of likes and dislikes and to classify them according to self-reported correctness	Out of 178 subject opinions taken, the correct classification had accuracy of 80.75%
Arnau, et al. [58]	N/A	Learning of arithmetical problem-solving via intelligent tutoring. Aimed at both expert and novice teachers	By implementing a search technique, the user’s answers were compared against a database of steps, and math problems were presented and solved on a step-by-step basis.	An accuracy of 82.14% was achieved in total out of the 75 subjects.
D’Mello and Graesser [59]	Direct measurement of expressions	Predicting student emotions such as boredom and engagement while using an ITS named Autotutor.	The research used the Autotutor, a validated ITS that helps students learn topics in science and computing via a mixed conversational dialog between the student and the tutor. There were 28 subjects in this study	The results showed that methods that detect affect in a text by identifying explicit articulations of emotion or by monitoring the valence of its content words are not always viable solutions.
Mohanan, et al. [60]	Face Detection	To examine the possibility of integrating an emotion detection system within an ITS	A class of subjects was observed while being taught by a teacher. The samples obtained from this method can be used to train the ITS. The ITS assessed the learner’s knowledge and present more challenging or less challenging tasks based on this assessment.	Capturing emotions by means of pictures and videos may not be an effective emotion recognition strategy. Students that are not very expressive, may not find the proposed ITS system effective. A more sophisticated way of identifying emotions can improve the quality of the research.
Njeru and Paracha [61]	Eye tracking	ITS with the use of model tracing and knowledge tracing, to assess the feasibility of using eye-tracking in learning.	Autotutor, a dialog and feedback-based learning software. Using Autotutor, 16 learners were asked to self-report their learning progress while being monitored for physiological signals	The paper explains how eye-tracking data can be used to detect learning behavior. However, it does not provide enough evidence to show feasibility of this method for emotion detection.

**Table 2:** Categories and methods of modern ITS.

ITS	Sensors Used	Classification	Recognized Emotions
Autotutor [110]	Video camera Pressure sensitive chair.	Extraction of eye pattern and posture, analysis of log files. Classifiers: C4.5 decision trees, neural networks, Naive Bayes, logistic regression, and nearest neighbor.	Frustrated, confused, bored, and neutral.
Cognitive BBN [116]	Video camera	Bayesian belief networks	Frustrated, confused, bored, happy, and interested.
Algebra Tutor [117]	N/A	Feature analysis such as activity history and student behavior. Classifiers: K* algorithm, J48 decision trees, step regression, Naive Bayes, REP-Trees.	Bored, concentrated, frustrated, and confused.
Affective learning companions [118]	mouse, posture chair, video camera, skin conductance bracelet	Hidden Markov Models, State Vector Machines, and Dynamic Bayesian Networks	Boredom, confusion, flow, and frustration.
Dynamic Bayesian Network (DBN) modeler [119]	N/A	Analysis of log files, interviews, and surveys. Through DBN emotions are modeled.	Enthusiastic, anxious, frustrated, bored, curious, confused, and focused.
Easy Eve [120]	Video Camera	Analysis of facial feature. Classifier: SVM.	Scared, surprised, smiling, laughing, disgusted, neutral, angry, and sad.
Arroyo’s ITS [120]	mouse, posture chair, camera, bracelet	Linear regression.	Confidence, frustration, excitement, interest.
EER-Tutor [121]	Video Camera	Extraction and tracking of facial features such as eyebrows and lips. Feature classification is carried out via comparing the neutral face with calculated distance for each facial feature.	Happy, angry, smiling, frustrated and neutral emotions.
Facial Features-based Tutor [115]	Video Camera	Extraction and tracking of facial feature and Regions of Interest (ROI). Classifiers: Fuzzy system, and neural network.	Scared, angry, disgusted, happy, sad, neutral, and surprised.
Gnu-Tutor [111]	Eye tracker	Analysis of log files, Gaze pattern extraction and eye tracking.	Disinterested, and bored.
InqITS [122]	N/A	Analysis of log files. Classifiers: step regression, J48 decision trees, JRip.	Concentrated, bored, confused, and frustrated.
INES-Tutor [123]	N/A	Analysis of the student’s difficulty of the task, activity level, previous progress, number of errors, severity of the error.	Confident, Worried, sad, and enthusiastic.
SPOKE [124]	Microphone	Extraction of acoustic-prosodic, lexical features and dialog features. Answer accuracy is assessed through semantic analysis.	Negative, positive, and neutral emotions.
MathSpring [125]	N/A	Analysis of log files, behavior patterns, and self-assessment reports. Classifier: linear regression.	Excited, inactive, confident, worried, interested, bored, satisfied, and frustrated.
Meta-Tutor [126]	Eye tracker	Extraction of gaze data features. Classifiers: SVM, random forests, logistic regression, and Naive Bayes.	Bored, curious, and interested.
PAT [127]	Video Camera	Analysis of log files, tracking, and extraction of facial feature points. Classification of motions are done via facial action coding.	Disappointed, happy, sad, satisfied, ashamed, grateful, and angry.
Prime-Climb [128]	Physiological sensors	Measuring heart rate, muscle activity, skin conductivity. Analyzing Biometric through unsupervised clustering.	Happy, sad (for the game), admiration, criticism (for PA), pride, shame (for himself).
VALERIE-Tutor [115]	Video camera, mic, physiological sensors	Measuring of skin conductivity, heart rate, extraction of speech, facial features, and analysis of mouse movement. Classifiers: Marquardt Back-propagation algorithm, nearest neighbor, linear discriminant function analysis.	Angry, surprised, sad, scared, amused, and frustrated.
Mavrikis’s ITS [129]	N/A	Analysis of log files. Classifier: J4.8 decision tree algorithm.	Frustrated, enthusiastic, confused, confident, bored, and happy.
